# Photodocumentation of the Development of Type I Posterior Glottic Stenosis after Intubation Injury

**DOI:** 10.1155/2015/504791

**Published:** 2015-02-01

**Authors:** Nelson Scott Howard, Travis L. Shiba, Julianna E. Pesce, Dinesh K. Chhetri

**Affiliations:** ^1^Department of Head and Neck Surgery, University of California-Los Angeles, 10833 Le Conte Avenue 62-132 CHS, Los Angeles, CA 90095, USA; ^2^Department of Otolaryngology-Head and Neck Surgery, Walter Reed Army Medical Center, Washington, DC, USA; ^3^Department of Surgery, Division of Head and Neck Surgery, San Antonio Military Medical Center, Fort Sam Houston, TX 78234-6200, USA

## Abstract

Bilateral vocal fold immobility may result from bilateral recurrent laryngeal nerve paralysis or physiologic insults to the airway such as glottic scars. The progression of mucosal injury to granulation tissue, and then posterior glottis stenosis, is an accepted theory but has not been photodocumented. This paper presents serial images from common postintubation injury to less common posterior glottic stenosis with interarytenoid synechia.

## 1. Introduction

Various pathologic conditions may be observed by the otolaryngologist following endotracheal intubation, including edema, ulcers, cartilage exposure, granulation tissue formation, or glottic stenosis. Management of the most common injuries is often conservative as granulation tissue and ulceration typically resolve spontaneously. The patients with progression to granulation formation are at risk of interarytenoid adhesion formation or early posterior glottic stenosis (PSG) and, therefore, should be closely monitored. While the majority of patients show some laryngeal injury after two days [1], less than 10% of patients intubated longer than five days show posterior glottic stenosis [2, 3].

Intubation trauma to the posterior commissure mucosa is theorized to cause ulceration, granulation, and then scar formation, which can fix the vocal cords [1, 2, 4–7]. PGS is associated with dysphonia, dyspnea, and tracheostomy dependence; early recognition and management of PGS are important, as advanced PGS types require more invasive treatment.

While numerous articles have described treatment methods for various stages of PGS based upon this theory, none has documented the progression from intubation injury to early posterior glottic stenosis [8, 9]. We present two patients with serial photographic evidence of intubation-related mucosal injury progressing to early posterior glottic stenosis.

## 2. Case Reports

Institutional review board (IRB) exemption was obtained. This retrospective review of two cases demonstrates early posterior glottic stenosis formation after intubation. Patients complained of progressive voice complaints and exhibited reduced vocal fold mobility. The photomicrographs shown here document the progressive changes over two months (patient 1) and 6 months (patient 2) after acute endotracheal tube injury. 

*Patient 1*. A 23-year-old male with a history of traumatic multilimb amputation injury was emergently intubated. He presented with symptoms of acute dysphonia, breathy voice, and vocal fatigue following extubation.

**Figure 1:**
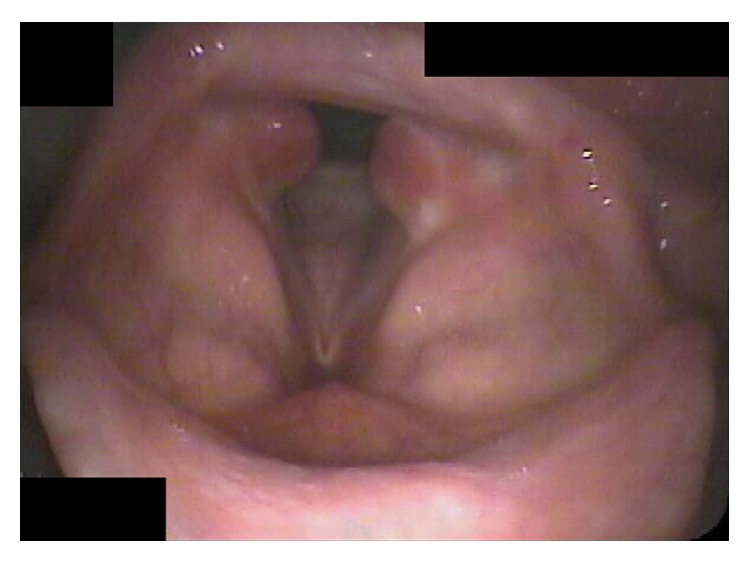
Early bilateral granulation tissue after intubation.

**Figure 2:**
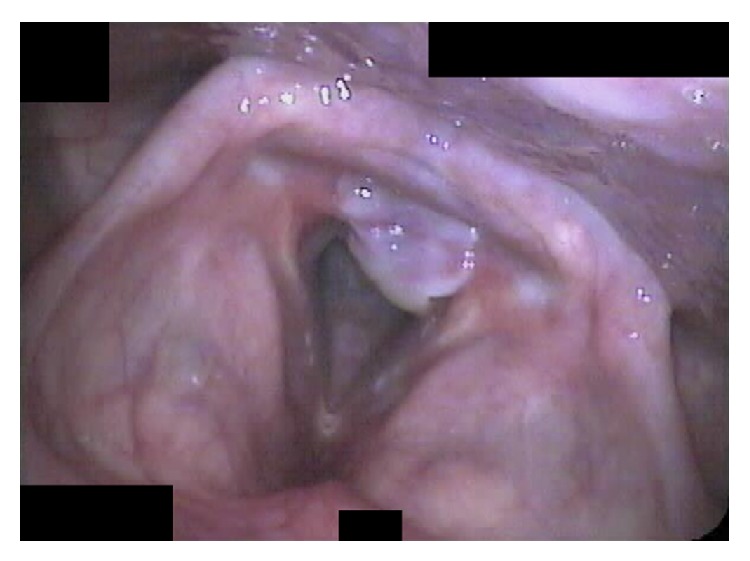
Formation of posterior glottic adhesion with fully mobile vocal folds.

**Figure 3:**
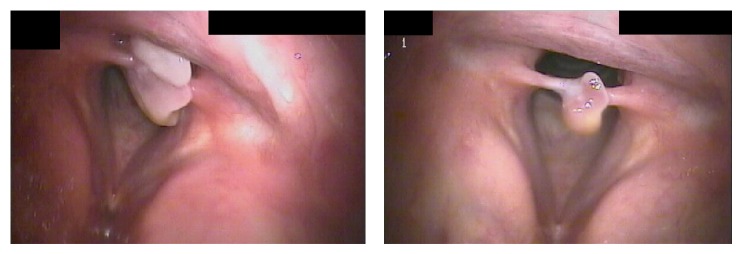
Mature posterior glottic granulation tissue and interarytenoid adhesion.

Early endoscopic examination of the patient after his hospitalization revealed granulation tissue overlying the mucosa of the medial surface of the bilateral vocal process of the arytenoid (Figure 1). Conservative management with inhaled steroids, antibiotics, and antireflux medications was instituted. Voice symptoms initially improved at three weeks despite the worsening appearance (Figure 2) and vocal fold motion was noted to be unrestricted at this time. However, worsening occurred by six weeks of management and included dyspnea on exertion, mild dysphagia, and a foreign body sensation in the throat; mature scar was noted at ten weeks (Figure 3) and patient was taken to the operating room for lysis of PSG. Symptoms resolved without complication for this patient.

**Figure 4:**
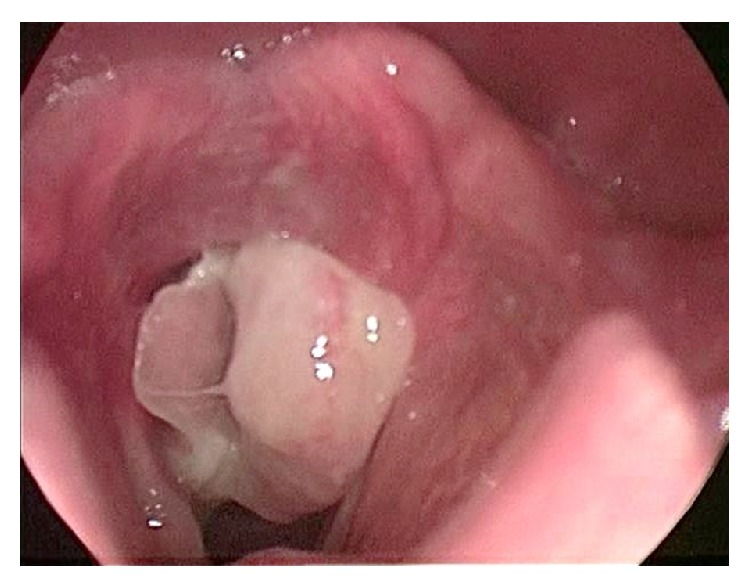
Postintubation granuloma.

**Figure 5:**
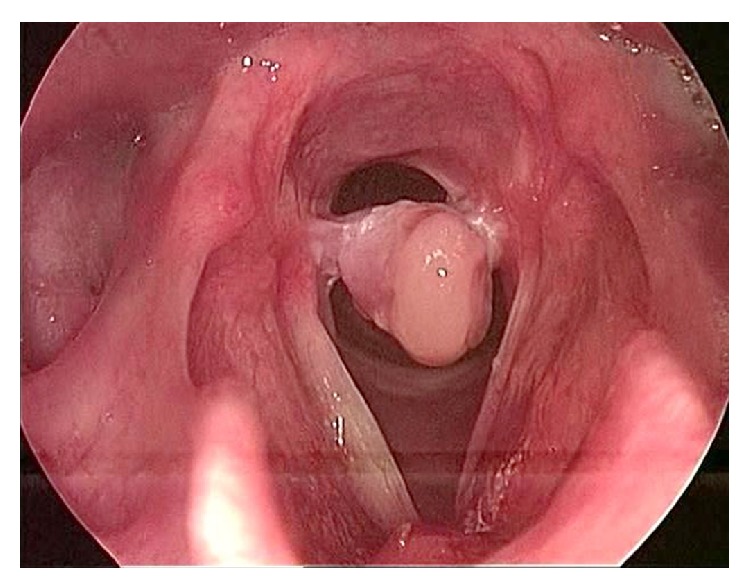
Post-Botox identification of interarytenoid adhesion.

*Patient 2*. A 30-year-old male with traumatic brain injury after an assault required prolonged intubation. Following extubation, the patient was found to have a granuloma expanding the posterior commissure (Figure 4). Transcutaneous Botox injection led to a reduction in granuloma size and an interarytenoid bridge was identified at follow-up four weeks later (Figure 5). Surgical intervention removed the granuloma and interarytenoid bridge but granulation tissue recurred. Additional Botox administration and continued observation followed. Repeat posterior interarytenoid bridge adhesion did not recur due to noncontact of the opposing granulation tissue.

## 3. Discussion

Endolaryngeal injuries continue to exist despite advances in endotracheal tube design and improvements in critical care management of patients on a mechanical ventilator. Mucosal injury typically occurs at one or more of the three most sensitive mucosal areas: the medial surfaces of the vocal process, the interarytenoid area, and the posterior subglottis along the inferior aspect of the cricoid cartilage [4].

While temporary dysphonia and mucosal injury to the glottis following tracheal intubation are quite common, glottic stenosis is less common and reportedly varies from 4 to 14%, with higher rates following prolonged intubation [3, 6]. Duration of intubation is associated with depth of mucosal injury, which is believed to be secondary to pressure necrosis leading to ischemia and edema. Increased granulation development is also associated with duration of intubation and can eventually lead to mucosal adhesions, scar tissue, joint immobility, and calcification [8, 10].

Factors such as local infection, inflammatory disorders, chronic disease states, hypotension, gastric reflux, tube mobility, and impaired pulmonary toilet are likely to be significant in the development of postintubation complications [7]. Additional factors that may influence long-term laryngeal sequelae of intubation include endotracheal tube size, obesity, tongue size, emergency intubation or traumatic intubation, requirement for cervical immobility, abnormal larynx anatomy, and poor patient healing characteristics.

Bogdassarian and Olson divided PGS into four categories of increasing severity [5]. Type 1 is described as the presence of interarytenoid adhesion without extension to the posterior commissure, leaving both anterior and posterior gaps. Type 2 describes extension to the posterior commissure; type 3 describes unilateral cricoarytenoid joint fixation; and type 4 describes bilateral cricoarytenoid joint fixation. Meyer and Wolf showed excellent prognosis for endoscopic treatment of PGS type 1 with greater than 80% decannulated and greater than 80% with voice improvement [9]. Other studies have reported high success rates with type 1 PSG treatment but treatment of PGS types 2–4 requires more extensive intervention [8, 9]. Methods used for treating the various stages of PGS have included arytenoidectomy, cordectomy, placement of a keel, micro-trap-door flaps, suture lateralization, open scar excision and mucosal advancement flap, open placement of posterior cricoid cartilage graft, laser lysis, and posterior mucosal flap [1, 3]. Management of endotracheal injuries requires endoscopic evaluation of the airway. In-office examination using topical lidocaine administered directly to the vocal folds enables the provider to inspect the posterior glottis when interarytenoid adhesions are suspected which may not be readily apparent due to postcricoid, interarytenoid, and arytenoid tissue prolapse into the airway.

Early edema and granulation tissue usually resolve within three weeks but the authors recommend that the airway should be monitored closely for resolution of symptoms. Nonoperative treatment recommendations vary considerably and are beyond the scope of this discussion. Development of changes in voice or dyspnea on exertion or other aerodigestive complaints a few weeks to months after intubation should prompt suspicion of restrictive posterior glottic stenosis. Immobile vocal cords may be best assessed during operative direct laryngoscopy with palpation of the arytenoid complex. Laryngeal electromyography may also be used to differentiate immobile vocal folds from paralysis or other neurologic disorders.

## 4. Conclusion

While postintubation injury is common, long-term complications are uncommon. This report demonstrates the development of PGS from postintubation granuloma, which has long been suspected but not documented. We emphasize the importance of serial examination of any patient who develops persistent voice alteration with findings of posterior glottic granulation tissue or with immobility or reduced mobility of the arytenoids after intubation. The photodocumentation shows the progression of common postintubation mucosal injury to much less common posterior glottic stenosis.
